# The impact of body composition variability on coagulation monitoring in patients on direct oral factor Xa inhibitors for treatment of venous thromboembolism

**DOI:** 10.3389/fcvm.2026.1773664

**Published:** 2026-03-13

**Authors:** Katharina Kurzmann-Guetl, Deborah R. Leitner, Thomas Gary, Alexander Avian, Andrea Beck, Jasmin Rabensteiner, Florian Prueller, Reinhard B. Raggam, Marianne Brodmann, Hermann Toplak

**Affiliations:** 1 Division of Angiology, Department of Internal Medicine, Medical University of Graz, Graz, Austria; 2 Lipid Clinic, Division of Endocrinology and Diabetology, Department of Internal Medicine, Medical University of Graz, Graz, Austria; 3 Medical Institute for Medical Informatics, Statistics and Documentation, Medical University of Graz, Graz, Austria; 4 Clinical Institute of Medical and Chemical Laboratory Diagnostics, Medical University Graz, Graz, Austria

**Keywords:** anticoagulation, body composition, obesity, oral factor Xa inhibitors, venous thromboembolism

## Abstract

**Background/objectives:**

Evidence for the first-line treatment of venous thromboembolism (VTE) by use of direct oral anticoagulants (DOACs) is based on stratification by body-mass index (BMI) and total body weight, but does not consider body composition – defined as the distribution of fat mass (FM) and fat-free mass (FFM). Variability in body composition exists among patients with normal BMI, but is anticipated to be markedly greater in the context of overweight and obesity.

**Methods:**

The BIARIVA prospective, single-center, cross-sectional study was conducted to assess whether body composition affects blood coagulation monitoring parameters in patients on therapeutic-dose anticoagulant treatment for VTE with oral factor (F.) Xa inhibitors rivaroxaban or edoxaban. Patients were qualified for inclusion into the study if they were categorized into one of the specified BMI categories; 18–25, 30–35, or > 35 kg/m^2^. Body composition was determined by two bioelectrical impedance analysis (BIA) methods. The primary endpoint was the association between body composition parameters and coagulation measurements in patients treated with rivaroxaban or edoxaban, assessed by correlation analysis.

**Results:**

Thirty-six patients on rivaroxaban and 35 patients on edoxaban were finally analyzed. The main finding in this study was a significant negative correlation observed in the edoxaban group for the absolute FFM with anti-F.Xa and plasma concentration peak levels (BIA method 1: r_s_ = −0.439, *p* = 0.008; BIA method 2: r_s_ = −0.431, *p* = 0.010) and with the absolute increase from trough to peak in plasma concentration levels (BIA method 1 and 2: r_s_ = −0.446, *p* = 0.007) and in anti-F.Xa levels (BIA method 1: r_s_ = −0.445, *p* = 0.007; BIA method 2: r_s_ = −0.444, *p* = 0.008). No significant correlations of body composition measures with coagulation parameters were found in the rivaroxaban group.

**Conclusions:**

This study suggests that body composition might influence specific coagulation measurements in patients treated with edoxaban, but further research is required to finally determine the role of body composition in this context and to evaluate clinical implications.

## 1 Introduction

Obesity is an established risk factor for venous thromboembolism (VTE), making decisions on anticoagulant treatment in this patient population an important clinical routine (1–3). Current guidelines recommend to use direct oral anticoagulants (DOACs) as the first-line treatment for most patients diagnosed with VTE (4–6). Safety and efficacy of the DOACs have been proven in large multi-centered, randomized-controlled trials, but a limited number of patients with obesity was included and stratification by body-mass index (BMI) is only available for some studies (7, 8). Furthermore, extensive meta-analyses and systematic reviews, which include real-world data, provide evidence supporting the safety and efficacy of DOACs in patients with VTE and atrial fibrillation (AF), even in individuals with a BMI exceeding 30 kg/m^2^ and 40 kg/m^2^ (9, 10). In 2021, the International Society of Thrombosis and Hemostasis (ISTH) published a guidance on the use of DOACs in patients with obesity, recommending to use standard-dose rivaroxaban or apixaban regardless of BMI and body weight, but suggesting not to use edoxaban or dabigatran in patients with a BMI > 40 kg/m^2^ or a total body weight > 120 kg (11). An Expert Consensus Panel recently addressed persisting uncertainties on DOAC's use in patients with obesity, and also provided a comprehensive update of the existing literature. While numerous studies further support the safe and effective use of rivaroxaban and apixaban even in cases of morbid obesity, robust clinical data for edoxaban and dabigatran are still lacking (12).

The classification of overweight and obesity as established by the World Health Organization (WHO) is based on the BMI, with definitions of BMI ≥ 25 kg/m^2^ for overweight and BMI ≥ 30 kg/m^2^ for obesity (13). Even if the BMI intends to classify body fat accumulation, it does not allow to assess body composition and, thus, to distinguish between fat mass (FM) and fat-free-mass (FFM) (14). While substantial variability in the distribution of FM and FFM exists even among patients with normal BMI, this variability is anticipated to be markedly greater in the context of obesity (15, 16). The Lancet Diabetes and Endocrinology Commission recently published a novel concept for the definition and diagnosis of obesity, recommending to confirm obesity by direct measurement of body fat or by collecting anthropometric parameters such as the waist circumference or the waist-to-hip ratio in addition to BMI (17). In recent times, the prevalence of overweight and obesity is still on the rise, and concerns regarding an adequate anticoagulant management with fixed-dose DOACs due to potential alterations in pharmacodynamics and pharmacokinetics particularly in patients with severe obesity persist (13, 18–20).

The objective of the BIARIVA study was to investigate the potential effects of body composition on coagulation monitoring parameters including anti-factor (F.) Xa activity and plasma concentration levels in VTE patients on anticoagulant treatment with oral F. Xa inhibitors rivaroxaban or edoxaban.

## 2 Methods

### 2.1 Study design

The BIARIVA study was conducted as a prospective, single-center, cross-sectional pilot study. Patient recruitment was performed between November 2018 and January 2020. Adult patients (≥ 18 years) with at least one prior event of VTE, treated with either full-dose rivaroxaban or full-dose edoxaban and eligible for bioimpedance measurements were enrolled. Full-dose anticoagulant treatment as recommended per the official drug information was rivaroxaban 20 mg once daily, edoxaban 60 mg once daily, and edoxaban 30 mg once daily for patients with a total body weight below 60 kg (21–24). Anticoagulant treatment was prescribed independently from the study participation and prior to the study inclusion, and all patients were under routine follow-up care for their pre-existing anticoagulant therapy at the local outpatient clinic. Furthermore, study participants were categorized based on their BMI into the following groups: “normal weight” group I BMI: 18–25 kg/m^2^, “moderate obesity” group II BMI: 30–35 kg/m^2^ and “severe obesity” group III BMI: > 35 kg/m^2^. Each BMI group was planned to require 10 patients for each substance, totaling a minimum of 60 patients. Given the exploratory nature of this prospective pilot study, no formal sample size calculation was performed. A sample size of 10 patients per BMI group was chosen to assess feasibility and preliminary associations between body composition parameters and anticoagulation measures. The primary endpoint was the association between body composition parameters and coagulation measurements in patients treated with rivaroxaban or edoxaban, assessed by correlation analysis. The study was conducted in accordance with the ethical principles of the Declaration of Helsinki and all applicable amendments laid down by the World Medical Assemblies and the International Conference of Harmonization Guidelines for good clinical practice. The study protocol was approved by the local ethics committee (protocol code 29-440 ex 16/17). All study participants provided written informed consent prior to participation.

### 2.2 Study visit

All prospectively enrolled patients completed a single study visit at the outpatient clinic, with two blood draws, anthropometric measurements, and evaluation of body composition. The observational period for each participant was approximately four hours. The first blood sample was drawn in a fasting condition and included routine laboratory parameters and specific coagulation monitoring parameters to determine trough levels of the respective anticoagulant, rivaroxaban or edoxaban. To allow for valid trough level measurement, all patients were instructed to have their last anticoagulant dose approximately 24 h prior to the study visit. The second blood draw was conducted ≥ two and ≤ four hours after drug intake of the respective substance, which corresponds to the time to peak for both substances (25–27). All patients on rivaroxaban were instructed to take their anticoagulant medication with some food as recommended by the official drug information (21, 22).

### 2.3 Coagulation measurements

Specific coagulation measurements included anti-F.Xa levels and direct plasma concentration levels for rivaroxaban, and anti-F.Xa levels for edoxaban. Venous blood samples for coagulation measurements were collected in Vacuette tubes of 3.5 mL volume (Greiner Bio-One, Kremsmünster, Austria) containing 3.8%-trisodium citrate. Samples were centrifuged at room temperature for 10 min following the local standard procedure and then stored at −20 °C until analysis. Anti-F.Xa levels were obtained using the validated, standard commercially available ready-to-use BIOPHEN Heparin LRT assay (HYPHEN BioMed, Neuilly-sur Oise, France) for chromogenic anti-F.Xa measurement of low-molecular weight heparin (LMWH) in human citrated plasma on the fully automated Atellica COAG 360 coagulation analyser system (Siemens Healthineers, Marburg, Germany). The use of a LMWH-calibrated anti-F.Xa measurement for determining the concentration of direct oral F.Xa inhibitors was based on several publications where calibration curves were adjusted for comparison with given ranges of linearity in measurements and lower and upper limits of detection, respectively (28–30). Plasma concentration levels were directly measured for rivaroxaban on an automated Atellica COAG 360 analyser (Siemens Healthineers, Marburg, Germany). For edoxaban, plasma concentration levels were calculated based on LMWH-calibrated anti-F.Xa levels following calibration by use of BIOPHEN edoxaban calibrator (HYPHEN Biomed, Neuilly-sur Oise, France): anti-F.Xa level (U/mL) × 148 = edoxaban plasma level (ng/mL). Besides measuring the trough and peak levels, the absolute increase in anticoagulant activity – defined as the absolute difference between trough and peak levels (*Δ*) – was calculated. Plasma concentration trough and peak levels in the BIARIVA study were compared to expected therapeutic ranges for patients on anticoagulant treatment for VTE using the 2021 Update of the International Council for Standardization in Haematology by Douxfils et al. (31). The expected ranges for the rivaroxaban cohort were 32 (6–239) ng/mL for trough and 215 (22–535) ng/mL for peak levels. For full-dose edoxaban (60 mg once daily), the expected levels were 19 (10–39) ng/mL for trough and 234 (149–317) ng/mL for peak measurements. For reduced-dose edoxaban (30 mg once daily), the expected levels were 16 (8–32) ng/mL for trough and 164 (99–225) ng/mL for peak measurements. These expected plasma concentrations published by Douxfils et al. are presented as mean (10th - 90th percentile) for rivaroxaban and as median (IQR) for edoxaban (31).

### 2.4 Anthropometric measurements

All participants were weighted in light clothing, without shoes and with an empty bladder on an electronic scale (seca mBCA 515, seca GmbH, Hamburg, Germany). The height was measured barefoot using the seca 360° length measuring device (seca 274, seca GmbH, Hamburg, Germany). Weight and height were recorded to the nearest 0.05 kg and 0.1 cm, respectively. The BMI of each participant was calculated as the ratio between the measured body weight (kg) and the square of the body height (m). Based on the calculated BMI, the participants were assigned to BMI categories: 18–25 kg/m^2^ (group I), 30–35 kg/m^2^ (group II) and > 35 kg/m^2^ (group III).

### 2.5 Body composition measurements

Two different bioelectrical impedance analysis (BIA) methods were used in all patients to determine body composition; the medical body composition analyser seca mBCA 515 (= BIA method 1; seca GmbH, Hamburg, Germany) and the BIACORPUS RX4000 (= BIA method 2; MEDI CAL HealthCare GmbH, Karlsruhe, Germany). The seca mBCA 515 device with its ease of use enables a tetrapolar measurement in an upright position, whereas the BIACORPUS RX4000 device can be used for bipolar measurements in a supine position. Both devices were used according to the manufacturers' manual (32, 33). FFM and FM were automatically calculated using the seca analytics 115 PC software (seca GmbH, Hamburg, Germany) and the BodyComp V.9.0 software (MEDI CAL HealthCare GmbH, Karlsruhe, Germany). FM and FFM are reported both in absolute terms (kg) and relative to total body weight (%), which is defined as the sum of FM and FFM.

### 2.6 Statistical analysis

Data are given as median and interquartile range (IQR) for continuous data, and as a frequency for categorical data. Baseline characteristics are compared using Mann Whitney U-Test for continuous variables and *χ*^2^ -Test for categorical data. With regard to the primary endpoint, associations between body composition and coagulation monitoring parameters were analyzed using Pearson's correlation coefficient (r) or Spearman's rank correlation coefficient (r_s_) as appropriate. Correlation coefficients are presented with 95% Confidence interval (95% CI). The magnitude of correlation coefficients is defined as poor <|0.3|, fair |0.3| to |0.5|, moderately strong |0.6| to |0.8| and very strong >|0.8| (34). An additional multivariable linear regression analysis was performed to investigate the influence of potential confounders including age, sex and creatinine, which was only added as an exploratory sensitivity analysis. Differences in bioimpedance measures obtained by two different BIA methods within BMI groups were analyzed using t-Test or Mann Whitney U-Test, and differences in measures between BMI categories for each substance were analyzed by Kruskal–Wallis test or ANOVA as appropriate (for *post hoc* pairwise comparison Bonferroni adjustment was used). The decision for parametric or non-parametric analysis was based on visual inspections of Q-Q plots and statistical tests (Shapiro–Wilk test). A *p*-value less than 5% was considered significant. For data analysis, IBM SPSS Statistics (IBM Corporation, Armonk, NY, USA) was used.

## 3 Results

In total, 80 patients were enrolled in the BIARIVA study, among them 41 patients on rivaroxaban treatment and 39 patients on edoxaban treatment. Seven patients did not meet the predefined BMI categories and were not included for the statistical analysis (four patients of the rivaroxaban group and three patients of the edoxaban group). Two patients were excluded due to an invalid body composition measurement (one patient in each group). Finally, a total of 71 patients **–** 36 in the rivaroxaban group and 35 in the edoxaban group **–** were analyzed. All rivaroxaban patients were on a full-dose treatment of 20 mg once daily. Among the edoxaban group, 31 patients received a full-dose treatment of 60 mg once daily, and four patients were on an adjusted dose of 30 mg for a body weight below 60 kg. The median age was 49.5 (33.7–61.9) among rivaroxaban patients and 58.0 (43.5–67.0) among edoxaban patients. Gender was equally distributed within the two groups, with 50% females in the rivaroxaban group and 45.7% in the edoxaban group. The median BMI was 33.0 (23.5–37.8) kg/m^2^ in rivaroxaban patients, and 33.7 (22.9–36.5) kg/m^2^ in edoxaban patients. Isolated deep vein thrombosis (DVT) was the most common indication for anticoagulant treatment in the rivaroxaban group (*n* = 14/36, 38.9%), whereas patients in the edoxaban group most frequently had a history of combined DVT and pulmonary embolism (PE) (*n* = 18/35, 51.4%). An overview of patient characteristics is presented in Table 1.

**Table 1:** Patient characteristics.

Characteristics	Rivaroxaban (*n* = 36)	Edoxaban (*n* = 35)	*p*-value
BMI group I (18–25 kg/m²)	*n* = 10/36 (27.8%; 13.9–41.7)	*n* = 11/35 (31.4%, 17.1–48.6)	0.90
BMI group II (30–35 kg/m²)	*n* = 12/36 (33.3%; 19.4–50.0)	*n* = 10/35 (28.6%; 14.3–45.7)	
BMI group III (> 35 kg/m²)	*n* = 14/36 (38.9%; 22.3–55.6)	*n* = 14/35 (40.0%; 22.9–57.1)	
BMI (kg/m^2^)	33.0 (23.5–37.8)	33.7 (22.9–36.5)	0.92
Age (years)	49.5 (33.7–61.9)	58.0 (43.5–67.0)	0.10
Female sex	*n* = 18/36 (50.0%; 33.3–66.7)	*n* = 16/35 (45.7%; 28.6–62.9)	0.72
Basic laboratory parameters			
Hemoglobin (g/dL)	14.1 (13.3–15.7)	13.8 (13.3–15.0)	0.41
Platelet count (10^9^/L)	227.5 (188.0–260.8)	243.0 (196.0–278.0)	0.35
WBC (10^9^/L)	6.16 (4.89–7.16)	5.99 (5.29–7.53)	0.75
Renal function*			
Creatinine (mg/dL)	0.83 (0.73–0.97)	0.92 (0.80–0.99)	0.06
GFR (mL/min)	92.36 (82.33–110.95)	82.29 (71.96–94.66)	0.012
Event			
PE	*n* = 12/36 (33.3%; 19.4–50.0)	*n* = 10/35 (28.6%; 14.3–45.7)	0.66
DVT	*n* = 14/36 (38.9%; 22.3–55.6)	*n* = 7/35 (20.0%; 8.6–34.3)	0.08
DVT + PE	*n* = 10/36 (27.8%; 13.9–41.7)	*n* = 18/35 (51.4%; 34.3–68.6)	0.041
Recurrent VTE^a^	*n* = 8/36 (22.2%; 11.1 36.1)	*n* = 6/35 (17.1%; 5.7–28.6)	0.59
Family history for VTE	*n* = 12/36 (33.3%; 19.4–50.0)	*n* = 11/35 (31.4%; 17.1–48.6)	0.86
VTE without any risk factor	*n* = 5/36 (13.9%; 2.8–27.8)	*n* = 5/35 (14.3%; 2.9–28.6)	0.96

Results are presented as frequency (%; 95%CI) or median (interquartile range).

BMI, body mass index; GFR, glomerular filtration rate; PE, pulmonary embolism; DVT, deep vein thrombosis; WBC, white blood cell count; VTE, venous thromboembolism.

^a^ Recurrent VTE was defined by more than one event of VTE documented in the medical history at the time of the inclusion into the study.

* Renal function parameters did not show a significant difference among BMI categories (creatinine_rivaroxaban_: *p* = 0.16, GFR_rivaroxaban_: *p* = 0.30; creatinine_edoxaban_: *p* = 0.14, GFR_edoxaban_: *p* = 0.25). Renal function parameters are not available for a single patient belonging to BMI group II due to non-performable laboratory measurements.

### 3.1 Body composition and coagulation analyses in rivaroxaban patients

Body composition measurements were obtained from all 36 patients on anticoagulant treatment with rivaroxaban from two different BIA systems (Table 2). The results for FM were significantly different within each BMI category when comparing measures obtained with the two methods, with a significantly higher FM when using BIA method 1 (*p* = 0.013 for BMI group I, *p* = 0.002 for BMI group II, *p* = 0.002 for BMI group III). The obtained coagulation monitoring measures within BMI categories are reported in Table 3.

**Table 2:** Overview of the body composition measurements for study participants.

Variables	Rivaroxaban (*n* = 36)	Edoxaban (*n* = 35)
BMI group I (18–25 kg/m^2^)	*n* = 10	*n* = 11
BMI (kg/m^2^)	21.0 (18.6–22.7)	22.3 (19.6–22.9)
BIA method 1 (seca mBCA 515)		
FM (kg)	15.8 (13.6–17.4)	15.5 (13.4–19.3)
FFM (kg)	39.3 (37.8–43.9)	45.3 (38.7–57.7)
BIA method 2 (BIACORPUS RX4000)		
FM (kg)	12.3 (9.7–15.0)	12.8 (11.1–16.6)
FFM (kg)	41.9 (40.3–45.3)	45.9 (40.1–62.0)
*Δ*FM_method 1 versus method 2 (kg)	2.4 (1.7–2.9)	2.6 (0.9–7.4)
*Δ*FM_method 1 versus method 2 (%)	4.3 (3.4–5.0)	3.9 (1.2–10.0)
BMI group II (30–35 kg/m^2^)	*n* = 12	*n* = 10
BMI (kg/m^2^)	31.9 (31.1–33.6)	33.5 (32.2–34.0)
BIA method 1 (seca mBCA 515)		
FM (kg)	38.5 (32.9–43.4)	42.2 (36.0–45.8)
FFM (kg)	63.1 (52.7–71.8)	65.0 (47.6–72.4)
BIA method 2 (BIACORPUS RX4000)		
FM (kg)	33.6 (30.2–39.2)	33.1 (26.2–38.4)
FFM (kg)	65.8 (55.9–78.6)	70.3 (56.4–78.3)
*Δ*FM_method 1 versus method 2 (kg)	4.2 (2.4–6.0)	6.2 (5.3–7.7)
*Δ*FM_method 1 versus method 2 (%)	4.4 (2.6–5.2)	6.2 (5.3–8.0)
BMI group III (> 35 kg/m^2^)	*n* = 14	*n* = 14
BMI (kg/m^2^)	39.2 (36.9–40.9)	37.6 (36.1–41.4)
BIA method 1 (seca mBCA 515)		
FM (kg)	53.6 (48.5–58.9)	48.0 (45.4–59.5)
FFM (kg)	73.7 (53.1–77.2)	73.2 (59.5–80.1)
BIA method 2 (BIACORPUS RX4000)		
FM (kg)	46.8 (44.2–57.8)	43.7 (41.3–55.7)
FFM (kg)	80.8 (54.4–85.6)	76.3 (58.2–86.6)
*Δ*FM_method 1 versus method 2 (kg)	5.0 (1.4–7.6)	4.0 (2.2–6.4)
*Δ*FM_method 1 versus method 2 (%)	4.1 (1.2–6.0)	3.4 (1.5–5.1)

Results are presented as median (interquartile range).

BIA, bioelectrical impedance analysis; BMI, body mass index; FM, fat mass; FFM, fat-free mass.

**Table 3:** Overview of coagulation measurements for study participants.

Variables	Rivaroxaban	Edoxaban
BMI group I (18–25 kg/m^2^)	*n* = 10	*n* = 11
Anti-F.Xa		
Trough (U/mL)	0.39 (0.27–0.50)	0.08 (0.05–0.32)
Peak (U/mL)	2.75 (2.41–2.92)	2.11 (1.51–2.44)
*Δ* (U/mL)	2.35 (1.91–2.67)	2.00 (1.43–2.40)
Plasma concentration		
Trough (ng/mL)	23.4 (12.0–26.6)	11.8 (7.4–47.4)
Peak (ng/mL)	232.9 (207.0–291.7)	312.3 (223.5–361.1)
*Δ* (ng/mL)	212.0 (176.7–282.1)	296.0 (211.6–355.2)
BMI group II (30–35 kg/m^2^)	*n* = 12	*n* = 10
Anti-F.Xa		
Trough (U/mL)	0.27 (0.13–0.36)	0.15 (0.09–0.16)
Peak (U/mL)	2.33 (1.80–2.70)	2.21 (1.57–2.46)
*Δ* (U/mL)	1.97 (1.58–2.45)	2.10 (1.50–2.29)
Plasma concentration		
Trough (ng/mL)	16.9 (6.5–30.5)	21.5 (13.3–23.7)
Peak (ng/mL)	176.3 (156.7–258.2)	327.1 (232.4–364.1)
*Δ* (ng/mL)	171.4 (132.4–229.2)	310.8 (222.0–338.9)
BMI group III (> 35 kg/m^2^)	*n* = 14	*n* = 14
Anti-F.Xa		
Trough (U/mL)	0.30 (0.17–0.73)	0.08 (0.05–0.13)
Peak (U/mL)	2.68 (2.30–2.92)	1.96 (1.55–2.22)
*Δ* (U/mL)	2.10 (1.61–2.63)	1.82 (1.47–2.17)
Plasma concentration		
Trough (ng/mL)	9.3 (5.0–50.4)	11.8 (7.4–19.2)
Peak (ng/mL)	244.9 (177.8–271.9)	289.3 (229.4–328.6)
*Δ* (ng/mL)	203.8 (150.3–239.8)	268.6 (217.6–321.2)

Results are presented as median (interquartile range).

BMI, body mass index; F., factor; *Δ*, increase from trough to peak levels.

When measurements of FM and FFM were used to evaluate the association between body composition and coagulation parameters, no significant correlation was detected among the rivaroxaban study group (Supplementary Table S1).

**Figure 1:**
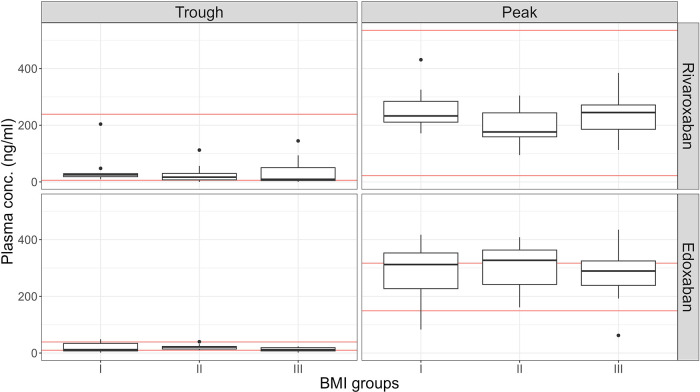
Distribution of plasma trough and peak levels with full-dose rivaroxaban or edoxaban referring to expected ranges of plasma levels among BMI categories. Expected therapeutic ranges of coagulation measures were 6–239 ng/mL for trough and 22–535 ng/mL for peak levels for the study cohort on rivaroxaban 20 mg once daily, and 10–39 ng/mL for trough measurements and 149–317 ng/mL for peak measurements for the study cohort on edoxaban 60 mg once daily. These expected plasma concentrations as reported by Douxfils et al. and are given as 10th – 90th percentile for rivaroxaban and as interquartile range (IQR) for edoxaban (31). Notably, four patients among the edoxaban cohort are not depicted in this figure as they received reduced-dose edoxaban (30 mg once daily) as recommended for a body weight below 60 kg and thus, have different expected on-therapy ranges. Lower and upper limits for the expected ranges are marked as red lines. The *Y*-axis presents plasma concentration levels in ng/mL and the *x*-axis shows BMI groups (BMI group I: 18–25 kg/m^2^, BMI group II: 30–35 kg/m^2^, BMI group III: > 35 kg/m^2^). The upper row shows distribution of plasma concentration levels at trough and peak for rivaroxaban patients and the lower row shows distribution of plasma concentration levels at trough and peak for edoxaban. BMI, body-mass index.

In the present study, 10 out of 36 patients (27.8%; 95%CI: 11.2–44.4) on rivaroxaban treatment did not meet the lower limit of the predefined therapeutic range for trough levels. Among them, six out of 10 were assigned to BMI group III, three to BMI group II and only one patient to BMI group I. No single patient exceeded the expected range for trough levels. For plasma peak levels, all patients (100%) met the expected therapeutic range. Plasma trough and peak levels among BMI categories in context with expected on-therapy ranges are illustrated in Figure 1.

### 3.2 Body composition and coagulation analyses in edoxaban patients

Body composition measurements were obtained from all 35 patients on edoxaban treatment using the two BIA systems (Table 2). In line with our observations in rivaroxaban patients, FM in patients of the edoxaban group was also significantly increased within each BMI category when measurements were performed with BIA method 1 (*p* = 0.008 for BMI group I, *p* = 0.005 for BMI group II, *p* = 0.002 for BMI group III).

**Figure 2:**
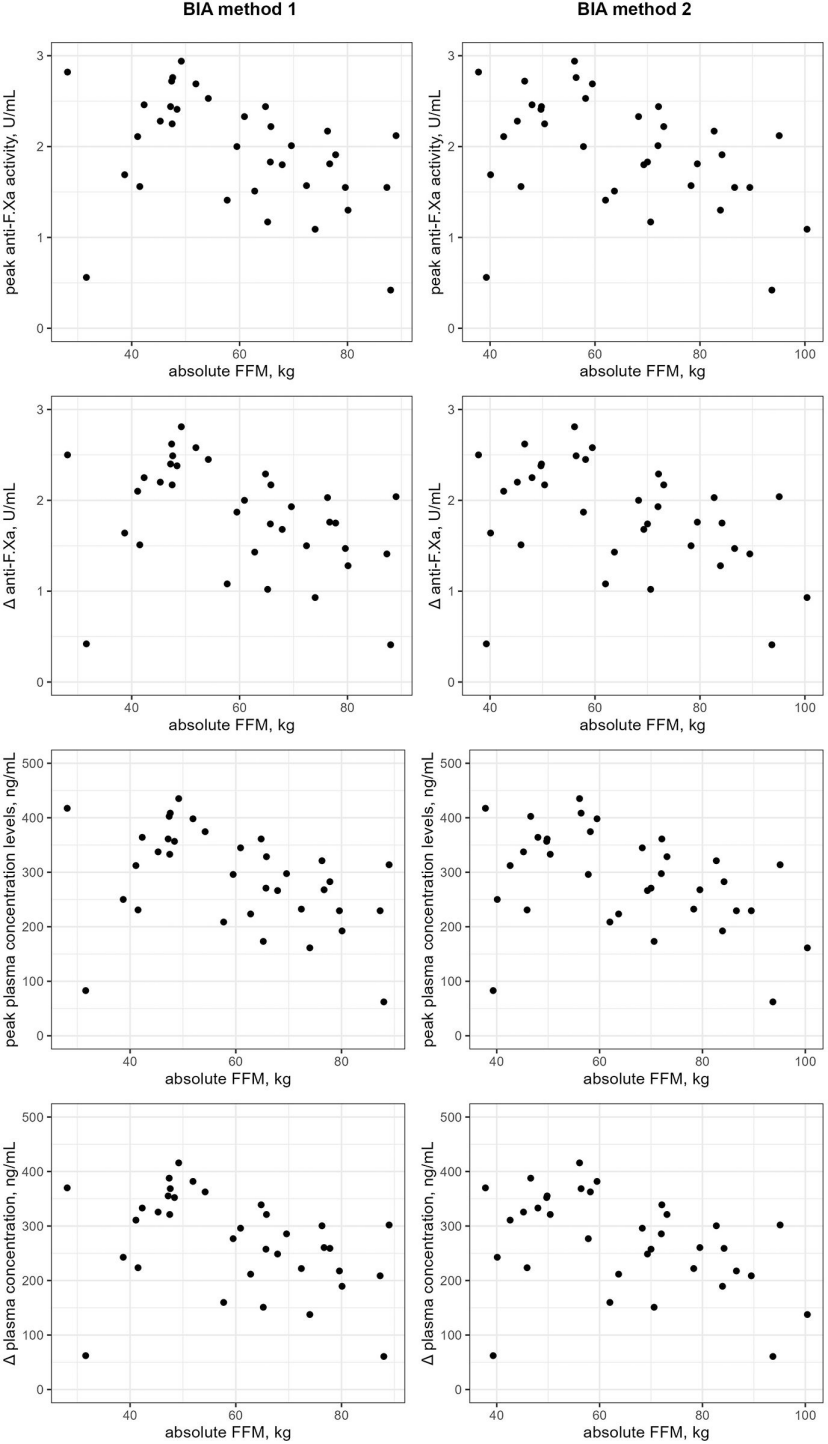
Scatterplots for statistically significant correlations between body composition measurements and coagulations parameters in the edoxaban study group. Statistically significant correlations were found in the edoxaban group using both BIA methods between the absolute FFM with peak anti-F.Xa activity, the anti-F.Xa absolute increase, peak plasma concentration levels and with the absolute increase of plasma concentration. BIA=bioelectrical impedance analysis; F., factor; FFM, fat-free mass; *Δ*, increase from trough to peak levels.

When body composition measures obtained with BIA method 1 were evaluated for their association with coagulation parameters, a significant negative correlation was revealed for the absolute FFM with peak anti-F.Xa activity (r_s_ = −0.439, 95%CI: −0.679 to −0.114, *p* = 0.008), the anti-F.Xa absolute increase (r_s_ = −0.445, 95%CI: −0.683 to −0.121, *p* = 0.007), peak plasma concentration levels (r_s_ = −0.439, 95%CI: −0.679 to −0.114, *p* = 0.008) and with the absolute increase of plasma concentration (r_s_ = −0.446, 95%CI: −0.684 to −0.123, *p* = 0.007). In concordance with the correlation analyses using BIA method 1, significant negative correlations of absolute FFM obtained by BIA method 2 with peak anti-F.Xa activity (r_s_ = −0.431, 95%CI: −0.674 to −0.104, *p* = 0.010), the anti-F.Xa absolute increase (r_s_ = −0.444, 95%CI: −0.682 to −0.120, *p* = 0.008), peak plasma concentration levels (r_s_ = −0.431, 95%CI: −0.674 to −0.104, *p* = 0.010) and with the absolute increase in plasma concentration levels (r_s_ = −0.446, 95%CI: −0.684 to −0.122, *p* = 0.007) were observed. However, the multivariable linear regression analysis, which confounded for age, sex and, renal function, did not show a significant association between the absolute FFM and coagulation parameters at peak and those indicating the absolute increase from trough to peak (Supplementary Table S2). No significant correlations with coagulation parameters were detected for absolute FM, percentage FM and percentage FFM obtained with both BIA methods. Scatterplots for significant correlations are shown in Figure 2. Full results for correlation analyses between body composition parameters and blood coagulation parameters in the edoxaban group are shown in Supplementary Table S1.

Nine out of 35 patients (25.7%, 95%CI: 11.4–40.0) did not meet the lower limit of the expected therapeutic range for edoxaban plasma trough levels. Among these patients, four were assigned to BMI group I and five to BMI group III. In four patients, trough levels were above the upper limit, three of them belonged to BMI group I and only one patient belonged to BMI group II. The predefined therapeutic range for edoxaban peak levels was not met for 19 out of 35 patients (54.3%, 95%CI: 37.1–71.4). While 17 of the 19 patients exceeded the upper limit of the expected range (seven from BMI group I, six from BMI group II, four from BMI group III), two of them were below the lower limit of the expected therapeutic range (one from BMI group I, and another one from BMI group III).

## 4 Discussion

The BIARIVA prospective, cross-sectional, single-center pilot study investigated the association between body composition and coagulation monitoring parameters in VTE patients treated with oral F.Xa inhibitors rivaroxaban or edoxaban. A significant correlation of FFM with coagulation parameters in the edoxaban study group was detected, but none in patients on treatment with rivaroxaban. In more detail, BIARIVA analyses in edoxaban treated patients revealed a significant negative correlation for absolute FFM with peak coagulation parameters and with the absolute increase in anticoagulant activity from trough to peak. The observed significant negative correlation indicates that lower absolute FFM was associated with increased anticoagulant activity and vice versa. An additional multivariable linear regression analysis was added as an exploratory sensitivity analysis. This analysis suggests that age and sex could be relevant confounders, warranting further investigation in a larger cohort. Furthermore, since anticoagulant activity was assessed solely through laboratory-based measurements of anti-F.Xa activity and plasma drug concentration levels, no conclusions can be drawn about its clinical significance, especially in relation to clinical outcomes. Consequently, the identified correlation should be regarded as a hypothesis-generating starting point for further investigation.

To strengthen the study's robustness and minimize method-specific biases, body composition measurements were conducted using two different BIA systems. The primary correlation analysis between body composition parameters and coagulation monitoring parameters consistently revealed statistically significant findings across measurements using both methods. The overall study results for FM were significantly different within each BMI category when comparing measures obtained with the two methods, with a significantly higher FM when using BIA method 1 (seca mBCA 515). This difference was∼5% and has been described previously (35). In general, BIA methods provide a non-invasive, widely accessible, and cost-effective approach that can be easily integrated into routine clinical practice. Nevertheless, despite the numerous advantages of BIA methods, non-invasive reference techniques such as dual-energy x-ray absorptiometry (DXA) are still considered the gold standard to determine FM and FFM, but are not routinely used in clinical practice (36, 37). Key limitations of BIA comprise that these methods rely on empirical equations and assumptions about constant tissue hydration, making it sensitive to hydration status, body geometry, and population characteristics, which can lead to systematic biases in estimated FM and FFM (37).

When comparing the pharmacodynamics and pharmacokinetics of the two DOACs, rivaroxaban and edoxaban, significant differences in their profiles are evident, despite both being highly selective, competitive and concentration-dependent inhibitors of F.Xa. Differences include various plasma protein binding (98% for rivaroxaban and 55% for edoxaban) and volume of distribution (50 L for rivaroxaban and 107 L for edoxaban) (38, 39). As both substances exhibit only moderate lipophilicity, no relevant accumulation in adipose tissue is expected (25, 40). Lipophilicity of drugs influences their distribution within the body and is quantified as log P, with higher values indicating greater lipophilicity. In this context, edoxaban not only has a lower lipophilicity compared to rivaroxaban (log P_edoxaban_ 0.90 to 1.61 vs. log P_rivaroxaban_ 1.74 to 1.90), but it also has the lowest lipophilicity among all approved DOACs (40). Therefore, a stronger association for both substances investigated in the BIARIVA study with FFM than with FM might be expected. From a pharmacokinetic perspective, a reduction in FFM may result in a smaller distribution volume and higher systemic exposure at a given dose, potentially contributing to increased plasma concentrations and anticoagulant activity. This may partially explain the findings of the present BIARIVA study, as there was a tendency of an association of FFM with coagulation peak parameters in the edoxaban study group. In general, absolute FFM is strongly dependent on overall body size. Furthermore, a reduced absolute FFM is typically observed in elderly, patients with chronic diseases (e.g., cancer) as well as in patients with malnutrition, sarcopenia, or prolonged physical inactivity. In contrast, an increased absolute FFM is typically observed in physically trained individuals and, in particular, in males, reflecting well-established physiological differences in muscle mass distribution. In the BIARIVA study, absolute FFM was lowest in BMI group I and highest in BMI group III. An increase in both absolute FM and absolute FFM was found with a rise in BMI, but the increase of FM was much more pronounced (FM varied between 28% and 36% in BMI group I of edoxaban patients and was at least 47% to 66% in BMI groups II and III).

When expected “on therapy” plasma drug concentration ranges were applied on peak and trough levels as obtained for the BIARIVA study participants, 27.8% of rivaroxaban patients and 25.7% of edoxaban patients had trough levels below the expected range. Regarding peak levels, all rivaroxaban patients met the expected range whereas two (5.7%) edoxaban patients were below the expected range. Interestingly, most rivaroxaban patients with trough levels below the expected “on-therapy” range were from BMI groups II or III (nine out of ten patients). Notably, the proportion of patients with trough levels below the expected range was most pronounced in patients with a BMI > 35 kg/m^2^ (six out of ten patients). Previous studies measuring rivaroxaban plasma concentration levels reported that 25%–50% of patients with obesity did not meet the expected “on-therapy” ranges (41, 42). This is in line with the results in the BIARIVA study for rivaroxaban trough levels, but not for peak levels. Notably, the expected on-therapy peak ranges published for patients receiving full-dose rivaroxaban for the diagnosis of VTE are extremely wide [215 (22–535 ng/mL), given as mean 10th – 90th percentile] as compared to peak ranges published for other indications such as AF (249 [164–343 ng/mL], given as mean [IQR]) (27). This very broad therapeutic window might explain our observation that 100% of rivaroxaban patients were within the expected therapeutic range for peak level measurements. For edoxaban, almost half of the study participants (48.6%) exceeded the expected therapeutic peak range. Of these, seven were from BMI group I, six from BMI group II, and four from BMI group III. In total, four participants in the BIARIVA study from BMI group I were receiving reduced-dose edoxaban because their body weight was below 60 kg. Due to the established reduction in drug exposure associated with reduced-dose edoxaban, specific “on-therapy” plasma drug concentration ranges have been defined (31, 43). When these specific ranges published for VTE were applied to the four patients in the BIARIVA study on reduced-dose edoxaban, most trough and peak levels measured were outside these predefined target ranges, above or also below. As for the very small sample size, further analysis was not feasible. However, the reduced dosing of edoxaban might also be considered a relevant confounder for the main correlation analysis performed in the BIARIVA study. Nevertheless, definite therapeutic targets are still unknown for DOACs. Currently available reference levels vary in literature and only represent “expected” or “on-therapy” ranges rather than therapeutic targets (11). Furthermore, no correlation has yet been reported between DOAC plasma concentration levels and clinical outcomes, hence the impact of these findings on daily clinical practice needs to be evaluated.

In light of available clinical data, a previously published analysis among patients receiving anticoagulant treatment with edoxaban for AF in the ENGAGE AF-TIMI 48 study population revealed that a higher BMI was significantly associated with a lower risk for stroke, systemic embolic events and death, but also with an increased risk for bleeding events, including major bleeding events and clinically relevant non-major bleeding events. However, no significant difference was detected for plasma concentration levels or for pharmacodynamic effects on F.Xa between patients with normal weight and those with obesity in the ENGAGE AF-TIMI 48 trial, but no detailed body composition profile was obtained (44).

In contrast to edoxaban, clinical data is available on the use of rivaroxaban specifically for the treatment of VTE in patients with obesity from *post hoc* analysis of the EINSTEIN trials and also from several retrospective, observational studies, demonstrating at least similar efficacy and safety for rivaroxaban when compared to vitamin K antagonists (45–51). Regarding pharmacokinetic investigations, rivaroxaban peak plasma concentrations in patients with extremes of body weight (> 120 kg) have been comparable to normal weight patients (52, 53).

### 4.1 Limitations of the study

Given the pilot nature of the study, the outcome is based on a small number of patients, with a relatively small sample size of obesity extremes (14.1% of the BIARIVA study population had a BMI > 40 kg/m^2^, and only one patient exceeded a BMI of 45 kg/m^2^). A consequence of this small group size is the low statistical power, which limits the ability to detect small effects and raises the potential issue of multiple testing. Considering the pilot study character, correction for multiple comparisons was not applied. The present study is also subject to inherent limitations related to its cross-sectional design with a single study visit. These include the inability to infer causal relationships from observed associations, susceptibility to selection bias, intra-individual variability over time, and restricted generalizability of the findings. Additional limitations include the single-center study design and the lack of a comprehensive assessment of concomitant medications that could interact with the DOACs. The relatively young median age of the study population may limit the generalizability of the findings, especially to older and multimorbid patient populations who constitute a substantial portion of individuals treated with DOACs. Lastly, body composition was exclusively assessed using BIA and not in addition to reference techniques like DXA or magnetic resonance imaging (MRI).

## 5 Conclusions

In this prospective, single-center, cross-sectional pilot study, no association between body composition and coagulation monitoring parameters was observed in patients on anticoagulant treatment with rivaroxaban, but some association for FFM with coagulation peak parameters was detected in patients treated with edoxaban. Further research is required especially in a larger study population to determine the role of body composition in the context of anticoagulant treatment and also to evaluate clinical outcomes.

## Data Availability

The raw data supporting the conclusions of this article will be made available by the authors, without undue reservation.
