# Supplementary material for: The impact of body composition variability on coagulation monitoring in patients on direct oral factor Xa inhibitors for treatment of venous thromboembolism
Source: Front Cardiovasc Med. 2026 Mar 13;13:1773664. doi: 10.3389/fcvm.2026.1773664 (PMC13021583; doi:10.3389/fcvm.2026.1773664)
Supplement: Supplementary file 1 [file Table1.docx]

# Supplementary Table S1: The correlation between body composition parameters and blood coagulation measures in patients on anticoagulant treatment with rivaroxaban or edoxaban

| **Rivaroxaban** | | | **BIA method 1**  **(seca mBCA 515)** | | | | | **BIA method 2**  **(BIACORPUS RX 4000)** | | | |
| --- | --- | --- | --- | --- | --- | --- | --- | --- | --- | --- | --- |
|  |  |  | **FM (kg)** | **FM (%)** | **FFM (kg)** | **FFM (%)** | | **FM (kg)** | **FM (%)** | **FFM (kg)** | **FFM (%)** |
| **Anti-F.Xa (trough; U/mL)** | r_s_ (95%CI) | | -0.174  (-0.483 – 0.174) | -0.094  (-0.418 – 0.251) | -0.185  (-0.492 – 0.163) | 0.094  (-0.251 – 0.418) | | -0.246  (-0.539 – 0.1) | -0.198  (-0.502 – 0.149) | -0.135  (-0.452 – 0.212) | 0.198  (-0.149 – 0.502) |
|  | sig | | 0.31 | 0.585 | 0.281 | 0.585 | | 0.148 | 0.247 | 0.434 | 0.247 |
| **Anti-F.Xa (peak; U/mL)** | r_s_ (95%CI) | | -0.067  (-0.395 – 0.277) | -0.013  (-0.349 – 0.326) | -0.102  (-0.425 – 0.244) | 0.013  (-0.326 – 0.349) | | -0.132  (-0.45 – 0.215) | -0.155  (-0.468 – 0.192) | -0.104  (-0.426 – 0.242) | 0.155  (-0.192 – 0.468) |
|  | sig | | 0.699 | 0.942 | 0.555 | 0.942 | | 0.442 | 0.366 | 0.547 | 0.366 |
| **Anti-F.Xa**  **(Δ; U/mL)** | r_s_ (95%CI) | | -0.093  (-0.417 – 0.252) | -0.027  (-0.361 – 0.313) | -0.129  (-0.447 – 0.218) | 0.027  (-0.313 – 0.361) | | -0.139  (-0.455 – 0.208) | -0.146  (-0.461 – 0.201) | -0.161  (-0.473 – 0.187) | 0.146  (-0.201 – 0.461) |
|  | sig | | 0.589 | 0.875 | 0.453 | 0.875 | | 0.417 | 0.395 | 0.349 | 0.395 |
| **Plasma_conc._ (trough; ng/mL)** | r_s_ (95%CI) | | -0.12  (-0.44 – 0.227) | -0.043  (-0.375 – 0.299) | -0.097  (-0.42 – 0.249) | 0.043  (-0.299 – 0.375) | | -0.185  (-0.492 – 0.163) | -0.117  (-0.438 – 0.229) | -0.048  (-0.379 – 0.295) | 0.117  (-0.229 – 0.438) |
|  | sig | | 0.485 | 0.802 | 0.575 | 0.802 | | 0.28 | 0.495 | 0.783 | 0.495 |
| **Plasma_conc._**  **(peak; ng/mL)** | r_s_ (95%CI) | | -0.053  (-0.388 – 0.295) | -0.012  (-0.353 – 0.332) | -0.121  (-0.445 – 0.231) | 0.012  (-0.332 – 0.353) | | -0.118  (-0.442 – 0.234) | -0.142  (-0.462 – 0.211) | -0.105  (-0.432 – 0.246) | 0.142  (-0.211 – 0.462) |
|  | sig | | 0.763 | 0.947 | 0.488 | 0.947 | | 0.501 | 0.416 | 0.549 | 0.416 |
| **Plasma_conc._**  **(Δ.; ng/mL)** | r_s_ (95%CI) | | -0.102  (-0.43 – 0.249) | -0.076  (-0.407 – 0.274) | -0.165  (-0.481 – 0.187) | 0.076  (-0.274 – 0.407) | | -0.151  (-0.469 – 0.202) | -0.18  (-0.492 – 0.173) | -0.178  (-0.491 – 0.175) | 0.18  (-0.173 - .,492) |
|  | sig | | 0.559 | 0.666 | 0.342 | 0.666 | | 0.388 | 0.3 | 0.305 | 0.3 |
| **Edoxaban** | | | **BIA method 1**  **(seca mBCA 515)** | | | | | **BIA method 2**  **(BIACORPUS RX 4000)** | | | |
|  |  |  | **FM (kg)** | **FM (%)** | **FFM (kg)** | | **FFM (%)** | **FM (kg)** | **FM (%)** | **FFM (kg)** | **FFM (%)** |
| **Anti-F.Xa (trough; U/mL)** | | r_s_ (95%CI) | 0.007  (-0.336 – 0.349) | 0.122  (-0.23 – 0.446) | -0.07  (-0.402 – 0.279) | | -0.122  (-0.446 – 0.23) | -0.146  (-0.465 – 0.207) | -0.08  (-0.411 – 0.27) | 0.009  (-0.334 – 0.35) | 0.08  (-0.27 – 0.411) |
|  |  | sig | 0.966 | 0.484 | 0.691 | | 0.484 | 0.403 | 0.648 | 0.96 | 0.648 |
| **Anti-F.Xa (peak; U/mL)** | | r_s_ (95%CI) | -0.115  (-0.44 – 0.237) | 0.282  (-0.067 – 0.569) | -0.439  (-0.679 – -0.114) | | -0.282  (-0.569 – 0.067) | -0.085  (-0.415 – 0.265) | 0.286  (-0.063 – 0.572) | -0.431  (-0.674 – -0.104) | -0.286  (-0.572 – 0.063) |
|  |  | sig | 0.512 | 0.101 | **0.008*** | | 0.101 | 0.627 | 0.096 | **0.010*** | 0.096 |
| **Anti-F.Xa**  **(Δ; U/mL)** | | r_s_ (95%CI) | -0.11  (-0.436 – 0.241) | 0.288  (-0.06 – 0.574) | -0.445  (-0.683 – -0.121) | | -0.288  (-0.574 – 0.06) | -0.064  (-0.398 – 0.284) | 0.306  (-0.041 – 0.587) | -0.444  (-0.682 – -0.12) | -0.306  (-0.587 – 0.041) |
|  |  | sig | 0.529 | 0.093 | **0.007*** | | 0.093 | 0.714 | 0.074 | **0.008*** | 0.074 |
| **Plasma_conc._ (trough; ng/mL)** | | r_s_ (95%CI) | 0.007  (-0.336 – 0.349) | 0.122  (-0.23 – 0.446) | -0.07  (-0.402 – 0.279) | | -0.122  (-0.446 – 0.23) | -0.146  (-0.465 – 0.207) | -0.08  (-0.411 – 0.27) | 0.009  (-0.334 – 0.35) | 0.08  (-0.27 – 0.411) |
|  |  | sig | 0.966 | 0.484 | 0.691 | | 0.484 | 0.403 | 0.648 | 0.96 | 0.648 |
| **Plasma_conc._**  **(peak; ng/mL)** | | r_s_ (95%CI) | -0.115  (-0.44 – 0.237) | 0.282  (-0.067 – 0.569) | -0.439  (-0.679 – -0.114) | | -0.282  (-0.569 – 0.067) | -0.085  (-0.415 – 0.265) | 0.286  (-0.063 – 0.572) | -0.431  (-0.674 – -0.104) | -0.286  (-0.572 – 0.063) |
|  |  | sig | 0.512 | 0.101 | **0.008*** | | 0.101 | 0.627 | 0.096 | **0.010*** | 0.096 |
| **Plasma_conc._**  **(Δ.; ng/mL)** | | r_s_ (95%CI) | -0.112  (-0.437 – 0.24) | 0.289  (-0.06 – 0.574) | -0.446  (-0.684 – -0.123) | | -0.289  (-0.574 – 0.06) | -0.065  (-0.398 – 0.284) | 0.307  (-0.04 – 0.587) | -0.446  (-0.684 – -0.122) | -0.307  (-0.587 – 0.04) |
|  |  | sig | 0.524 | 0.093 | **0.007*** | | 0.093 | 0.711 | 0.073 | **0.007*** | 0.073 |

Results are presented as correlation coefficients (corr) and their significance levels (sig). 95% confidence intervals (CI) are provided for correlation coefficients. Statistically significant results are marked with an asterix (*).

Abbreviations: BIA = bioelectrical impedance analysis; conc. = concentration; F. = factor; FM = fat mass; FFM = fat-free mass; Δ = increase from trough to peak levels.
