# Supplementary material for: The impact of body composition variability on coagulation monitoring in patients on direct oral factor Xa inhibitors for treatment of venous thromboembolism
Source: Front Cardiovasc Med. 2026 Mar 13;13:1773664. doi: 10.3389/fcvm.2026.1773664 (PMC13021583; doi:10.3389/fcvm.2026.1773664)
Supplement: Supplementary file 2 [file Table2.docx]

# Supplementary Table S2: Linear regression analysis results for different outcomes and predictors adjusted for age, sex and creatinine in patients on anticoagulant treatment with rivaroxaban or edoxaban

|  |  |  | **Rivaroxaban** | | **Edoxaban** | |
| --- | --- | --- | --- | --- | --- | --- |
|  | **Predictor** |  | **BIA method 1**  **(seca mBCA 515)** | **BIA method 2**  **(BIACORPUS RX 4000)** | **BIA method 1**  **(seca mBCA 515)** | **BIA method 2**  **(BIACORPUS RX 4000)** |
| **Anti-F.Xa**  **(trough; U/mL)** | FM, kg | p-value  β  95%CI (β) | 0.757  -0.001  (-0.009 – 0.007) | 0.639  -0.002  (-0.009 – 0.006) | 0.02  -0.002  (-0.003 - 0) | 0.009  -0.002  (-0.003 – -0.001) |
|  | FM, % | p-value  β  95%CI (β) | 0.865  0.001  (-0.013 – 0.016) | 0.874  -0.001  (-0.013 – 0.012) | 0.029  -0.003  (-0.006 – 0) | 0.003  -0.003  (-0.006 – -0.001) |
|  | FFM, kg | p-value  β  95%CI (β) | 0.347  -0.006  (-0.02 – 0.007) | 0.547  -0.004  (-0.017 – 0.009) | 0.034  -0.003  (-0.006 – 0) | 0.173  -0.002  (-0.004 – 0.001) |
|  | FFM, % | p-value  β  95%CI (β) | 0.865  -0.001  (-0.016 – 0.013) | 0.874  0.001  (-0.012 – 0.013) | 0.029  0.003  (0 – 0.006) | 0.003  0.003  (0.001 – 0.006) |
| **Anti-F.Xa**  **(peak; U/mL)** | FM, kg | p-value  β  95%CI (β) | 0.3  -0.01  (-0.028 – 0.009) | 0.197  -0.012  (-0.03 – 0.006) | 0.737  0.002  (-0.01 – 0.015) | 0.59  0.003  (-0.009 – 0.015) |
|  | FM, % | p-value  β  95%CI (β) | 0.88  -0.003  (-0.038 – 0.033) | 0.244  -0.017  (-0.047 – 0.012) | 0.796  0.003  (-0.02 – 0.026) | 0.486  0.007  (-0.013 – 0.027) |
|  | FFM, kg | p-value  β  95%CI (β) | 0.032  -0.034  (-0.065 – -0.003) | 0.128  -0.023  (-0.054 – 0.007) | 0.787  0.003  (-0.02 – 0.027) | 0.902  -0.001  (-0.022 – 0.02) |
|  | FFM, % | p-value  β  95%CI (β) | 0.8  0.003  (-0.033 – 0.038) | 0.244  0.017  (-0.012 – 0.047) | 0.796  -0.003  (-0.026 – 0.02) | 0.486  -0.007  (-0.027 – 0.013) |
| **Anti-F.Xa**  **(Δ; U/mL)** | FM, kg | p-value  β  95%CI (β) | 0.307  -0.008  (-0.025 – 0.008) | 0.219  -0.01  (-0.026 – 0.006) | 0.526  0.004  (-0.008 – 0.016) | 0.383  0.005  (-0.007 – 0.017) |
|  | FM, % | p-value  β  95%CI (β) | 0.803  -0.004  (-0.035 – 0.027) | 0.215  -0.016  (-0.042 – 0.01) | 0.591  0.006  (-0.017 – 0.029) | 0.285  0.01  (-0.009 – 0.03) |
|  | FFM, kg | p-value  β  95%CI (β) | 0.052  -0.028  (-0.056 - 0) | 0.154  -0.019  (-0.047 – 0.008) | 0.588  0.006  (-0.017 – 0.029) | 0.961  0.001  (-0.02 – 0.021) |
|  | FFM, % | p-value  β  95%CI (β) | 0.803  0.004  (-0.027 – 0.035) | 0.215  0.016  (-0.01 – 0.042) | 0.591  -0.006  (-0.029 – 0.017) | 0.285  -0.01  (-0.03 – 0.009) |
| **Plasma_conc._**  **(trough; ng/mL)** | FM, kg | p-value  β  95%CI (β) | 0.742  -0.114  (-0.816 – 0.588) | 0.658  -0.152  (-0.846 – 0.542) | 0.02  -0.261  (-0.477 – -0.045) | 0.009  -0.281  (-0.487 – -0.075) |
|  | FM, % | p-value  β  95%CI (β) | 0.835  0.135  (-1.18 – 1.45) | 0.987  -0.009  (-1.135 – 1.117) | 0.029  -0.457  (-0.863 – -0.051) | 0.003  -0.507  (-0.833 – -0.181) |
|  | FFM, kg | p-value  β  95%CI (β) | 0.334  -0.597  (-1.839 – 0.645) | 0.481  -0.41  (-1.584 – 0.764) | 0.034  -0.446  (-0.857 – -0.036) | 0.173  -0.264  (-0.651 – 0.123) |
|  | FFM, % | p-value  β  95%CI (β) | 0.835  -0.135  (-1.45 – 1.18) | 0.987  0.009  (-1.117 – 1.135) | 0.029  0.457  (0.051 – 0.863) | 0.003  0.507  (0.181 – 0.833) |
| **Plasma_conc._**  **(peak; ng/mL)** | FM, kg | p-value  β  95%CI (β) | 0.267  -0.902  (-2.532 – 0.727) | 0.191  -1.049  (-2.648 – 0.551) | 0.737  0.308  (-1.548 – 2.165) | 0.59  0.481  (-1.323 – 2.285) |
|  | FM, % | p-value  β  95%CI (β) | 0.672  -0.649  (-3.752 – 2.453) | 0.238  -1.532  (-4.132 – 1.068) | 0.796  0.441  (-3.01 – 3.893) | 0.486  1.012  (-1.919 – 3.943) |
|  | FFM, kg | p-value  β  95%CI (β) | 0.069  -2.609  (-5.43 – 0.213) | 0.181  -1.821  (-4.537 – 0.895) | 0.787  0.464  (-3.008 – 3.937) | 0.902  -0.19  (-3.319 – 2.939) |
|  | FFM, % | p-value  β  95%CI (β) | 0.672  0.649  (-2.453 – 3.752) | 0.238  1.532  (-1.068 – 4.132) | 0.796  -0.441  (-3.893 – 3.01) | 0.486  -1.012  (-3.943 – 1.919) |
| **Plasma_conc._**  **(Δ.; ng/mL)** | FM, kg | p-value  β  95%CI (β) | 0.287  -0.788  (-2.273 – 0.696) | 0.219  -0.897  (-2.356 – 0.563) | 0.526  0.569  (-1.245 – 2.384) | 0.383  0.761  (-0.997 – 2.519) |
|  | FM, % | p-value  β  95%CI (β) | 0.574  -0.784  (-3.6 – 2.031) | 0.196  -1.523  (-3.877 – 0.831) | 0.591  0.898  (-2.479 – 4.276) | 0.285  1.519  (-1.329 – 4.366) |
|  | FFM, kg | p-value  β  95%CI (β) | 0.126  -2.012  (-4.621 – 0.597) | 0.257  -1.411  (-3.902 – 1.081) | 0.588  0.911  (-2.488 – 4.309) | 0.961  0.074  (-3 – 3.148) |
|  | FFM, % | p-value  β  95%CI (β) | 0.574  0.784  (-2.031 – 3.6) | 0.196  1.523  (-0.831 – 3.877) | 0.591  -0.898  (-4.276 – 2.479) | 0.285  -1.519  (-4.366 – 1.329) |

P-values for predictors and regression coefficients (β) with 95% confidence intervals (CI) are shown.

Abbreviations: BIA = bioelectrical impedance analysis; conc. = concentration; F. = factor; FFM = fat-free mass; FM = fat mass; Δ = increase from trough to peak levels.
